# xoxF-linked methanol oxidation signals recur across wetland metagenomes

**DOI:** 10.1128/spectrum.00889-26

**Published:** 2026-09-01

**Authors:** Sukhjit Kaur, Archana Anand

**Affiliations:** 1 Department of Biology, San Francisco State University 117250 , San Francisco, California, USA; Luonnonvarakeskus, Oulu, Finland; Oak Crest Institute of Science, Pasadena, California, USA; University of the Punjab, Lahore, Pakistan

**Keywords:** wetland metagenomes, xoxF, methanol oxidation, methylotrophy, lanthanide-dependent methanol dehydrogenase, methane cycling

## Abstract

Wetlands are globally important methane-cycling ecosystems, but methanol oxidation remains less frequently emphasized than methane oxidation in community-level metagenomic analyses. Recent studies have highlighted the ecological importance of the lanthanide-dependent methanol dehydrogenase XoxF, yet wetland-focused xoxF literature remains comparatively limited relative to marine, freshwater, and other environmental systems. Here, we screened wetland-associated records in IMG/M using methane-metabolism and enzyme-centered queries focused on EC 1.1.2.10/xoxF and related methanol-oxidation annotations. Wetland-associated data sets repeatedly returned xoxF-linked annotations. We then examined a representative data set, a Wetland Surface Sediment combined assembly, to assess the relative representation of methanol-oxidation-associated annotations. In this co-assembly, xoxF/EC 1.1.2.10 was the most frequently recovered methanol-oxidation-associated annotation in the queried annotation space, with 1,996 genes, compared with 428 genes assigned to mxa-like EC 1.1.2.7 functions and 235 genes assigned to mdo/EC 1.1.99.37. KEGG-linked organism context included canonical methanotrophic and methylotrophic genera, including *Methylococcus, Methylomonas, Methylotuvimicrobium, Methylovulum*, and *Methylophaga*, while also extending into broader environmental Proteobacteria. Together, these data indicate that xoxF-linked methanol oxidation annotations recur across wetland metagenomes and are strongly represented in a representative wetland surface sediment co-assembly. Because this study relies on database annotations rather than organism-resolved pathway reconstruction or activity measurements, the results are best interpreted as community-level functional potential rather than evidence of xoxF-mediated activity, flux, or ecological dominance. These findings support wetlands as an underexamined but relevant setting for future clade-resolved, genome-resolved, and activity-resolved studies of xoxF-associated methanol oxidation.

**IMPORTANCE:**

Wetlands play a major role in Earth’s methane cycle, but studies of wetland microbes often focus more on methane itself than on the downstream step of methanol oxidation. Our study shows that wetland metagenomes repeatedly contain strong signals for xoxF, a gene linked to lanthanide-dependent methanol oxidation. In a representative wetland surface sediment co-assembly, xoxF-associated annotations were much more abundant than classical methanol dehydrogenase annotations, suggesting that this pathway may be especially important in wetland microbial communities. Because most previous xoxF studies have focused on marine, freshwater, or other non-wetland systems, these findings highlight wetlands as an underexplored setting for methanol-processing metabolism. This work provides a foundation for future studies connecting these signals to specific microbes, environmental conditions, and methane-cycling processes in wetlands.

## OBSERVATION

Wetlands are globally important sites of methane production and methane turnover, making them key habitats for understanding microbial pathways that influence carbon cycling and greenhouse gas exchange. Recent syntheses identify wetlands as the largest single natural source of atmospheric methane, contributing roughly 25%–35% of total global methane sources, with commonly cited global wetland emissions in the range of about 100–230 Tg CH₄ year⁻¹; an ensemble estimate for 2010–2020 placed mean wetland emissions at 158 ± 24 Tg CH₄ year⁻¹ (1, 2). Methane turnover within wetlands is also substantial because aerobic methane oxidation can consume a significant fraction of produced methane before emission although the magnitude of this fraction varies widely across wetland types, seasons, and redox conditions. For example, in a freshwater wetland, seasonal aerobic methane oxidation ranged from 15% to 76% of the potential diffusive methane flux, and on an annual basis, approximately 43% of methane diffusing into the oxic zone was oxidized before reaching the atmosphere (3). Although methane oxidation has received sustained attention in wetland microbiology, the downstream step of methanol oxidation is less often foregrounded in community-level metagenomic studies. In methanotrophs and other methylotrophs, methanol oxidation links methane-derived carbon to formaldehyde production, downstream assimilation pathways, and respiratory energy metabolism (4, 5).

Historically, environmental studies of bacterial methanol oxidation emphasized the calcium-dependent methanol dehydrogenase MxaFI, often using mxaF as a functional marker. Over the past decade, however, the lanthanide-dependent methanol dehydrogenase XoxF has emerged as an ecologically important alternative. Representative studies summarized in Table 1 show that xoxF-linked methanol oxidation has been documented across coastal marine, pelagic marine, freshwater, and other environmental systems, while comparatively fewer studies have focused directly on wetlands. Environmental surveys showed that xoxF is widespread in coastal marine systems, and marker-based studies in pelagic marine and freshwater environments demonstrated that xoxF-centered approaches can recover methylotrophic populations that would be underrepresented or missed by mxaF-only screening (6, 7). Comparative studies further showed that XoxF clades differ in distribution and biochemical properties, indicating that xoxF-rich data sets may contain functionally and ecologically distinct methylotrophic assemblages rather than a single uniform xoxF-containing group (8). Mechanistic studies also support the biological importance of XoxF beyond detection in sequence data sets. In *Methylomicrobium buryatense*, lanthanides increased xoxF expression, reduced mxa expression, and supported XoxF as the predominant methanol dehydrogenase under tested conditions, helping establish the now widely discussed lanthanide switch (4).

**TABLE 1:** Literature on xoxF-linked methanol oxidation across environmental and experimental systems

Study	System/ecosystem	Genes/pathways emphasized
Taubert et al. (7)	Coastal marine environments	xoxF, methanol oxidation
Ramachandran et al. (6)	Pelagic marine, freshwater, and estuarine waters	xoxF4, xoxF5, mxaF
Huang et al. (8)	Broad comparative methylotroph data set	xoxF4, xoxF5
Chu and Lidstrom (4)	*Methylomicrobium buryatense* isolate	xoxF, mxaFI
Roszczenko-Jasińska et al. (9)	Lanthanide-rich shale rock	xoxF
Daszczyńska et al. (10)	Lanthanide-rich deep underground environment/mine microbial mat	xoxF clades, methanol metabolism
Yun et al. (5)	Zoige alpine wetland	xoxF, mxaF, methane oxidation pathways
Gwak et al. (11)	Wetland-associated methane/sulfur-oxidizing microorganism from redox interfaces	Methane oxidation, sulfur oxidation, XoxF-type alcohol dehydrogenases
Mineo et al. (12)	Soil-associated symbiotic bacterium	XoxF and the Calvin-Benson cycle
Yang et al. (13)	Review of rare-earth-element-dependent biomolecules across biological systems	XoxF-type methanol dehydrogenases, lanthanide-associated proteins, rare-earth-element-dependent metabolism

The ecological distribution of xoxF-like genes is also broader than canonical methanotrophs and methylotrophs alone. Recent synthesis of rare-earth-element-dependent biomolecules highlights XoxF-type methanol dehydrogenases as part of a wider set of lanthanide-associated proteins and metabolisms, underscoring that xoxF-like annotations can occur across diverse bacterial lineages and environmental contexts (13). This broader distribution strengthens the ecological relevance of xoxF-centered screening, but it also requires caution: annotation-based recovery of xoxF cannot by itself distinguish methanotrophic from non-methanotrophic sources, resolve xoxF clades, or establish pathway activity.

Despite this broader progress, wetland-focused literature remains comparatively limited. One important wetland comparator is the Zoige alpine wetland study, which recovered active methanotroph MAGs containing both xoxF and mxaF, demonstrating the relevance of these markers in wetlands while also underscoring how limited the wetland-specific evidence base remains (5). Because wetlands are methane-cycling ecosystems, xoxF-linked methanol oxidation in these systems should be interpreted in relation to upstream methane oxidation, including the possibility that some xoxF signals derive from methanotrophs where methanol oxidation occurs downstream of methane oxidation. In this study, we, therefore, used IMG/M annotation-centered screening to ask whether xoxF-linked methanol-oxidation annotations recur across wetland-associated metagenomic records and how strongly they are represented relative to selected mxa-like and mdo-associated methanol-oxidation annotations in a representative wetland surface sediment co-assembly.

## MATERIALS AND METHODS

To examine xoxF-linked methanol oxidation potential in wetlands, a search was performed on the Joint Genome Institute IMG/M Integrated Microbial Genomes and Microbiomes database using methane-metabolism and enzyme-based queries centered on EC 1.1.2.10 (xoxF). Search terms included “methane metabolism,” “EC 1.1.2.10,” “xoxF,” “K23995,” “EC 1.1.2.7,” “mxaF,” “mxaI,” and “EC 1.1.99.37.” Wetland-associated records returned recurrent xoxF-linked annotations, motivating a focused analysis of a representative wetland data set, the Wetland Surface Sediment combined assembly (3300075153). From this record, methane-metabolism results and associated exported gene tables were examined to compare the prevalence of xoxF, classical methanol dehydrogenase-associated genes (mxaF/mxaI), and related methanol-oxidation annotations. Exported hits were then organized by gene class and scaffold context to assess the distribution of methanol-oxidation functions across the co-assembly.

Because this data set is a combined metagenomic co-assembly, annotation patterns were interpreted as community-level functional potential rather than organism-resolved pathway assignment. This analysis relied on existing IMG/M, EC, and KEGG annotations and did not include *de novo* sequence reannotation, hidden Markov model validation, xoxF clade classification, MAG reconstruction, transcriptomic analysis, proteomic analysis, or activity-based validation. Therefore, reported counts should be interpreted as database-derived annotation counts rather than direct measures of gene activity, enzyme abundance, methanol-oxidation flux, or ecological dominance.

## RESULTS AND DISCUSSION

Wetland-associated IMG/M screening repeatedly recovered annotations linked to EC 1.1.2.10/xoxF, indicating that xoxF-linked methanol oxidation annotations recur across wetland metagenomic data sets. This pattern is notable because, as summarized in Table 1, much of the published xoxF literature has focused on marine, freshwater, estuarine, and other non-wetland systems, with comparatively fewer wetland-focused examples. The repeated recovery of xoxF-linked annotations across wetland data sets, therefore, supports wetlands as an underexamined but relevant setting for xoxF-associated methanol oxidation potential.

To examine this signal in more detail, we focused on a representative data set, the Wetland Surface Sediment combined assembly (3300075153). Because this record is a combined metagenomic co-assembly, the resulting patterns are best interpreted as evidence of community-level functional potential rather than direct organism-level pathway assignment. Within the methane-metabolism output for this co-assembly, xoxF/EC 1.1.2.10 was the most frequently recovered methanol-oxidation-associated annotation in the queried annotation space, with 1,996 genes, compared with 428 genes assigned to mxa-like methanol dehydrogenase functions (EC 1.1.2.7) and 235 genes assigned to mdo (EC 1.1.99.37) (Fig. 1). The mdo category represents nicotinoprotein methanol dehydrogenases, an additional methanol-oxidation-associated enzyme group distinct from classical MxaFI-type and lanthanide-dependent XoxF-type methanol dehydrogenases. Thus, xoxF-linked annotations exceeded the mxa-like annotation count by more than fourfold and the mdo annotation count by more than eightfold in this representative wetland co-assembly.

**Fig 1:**
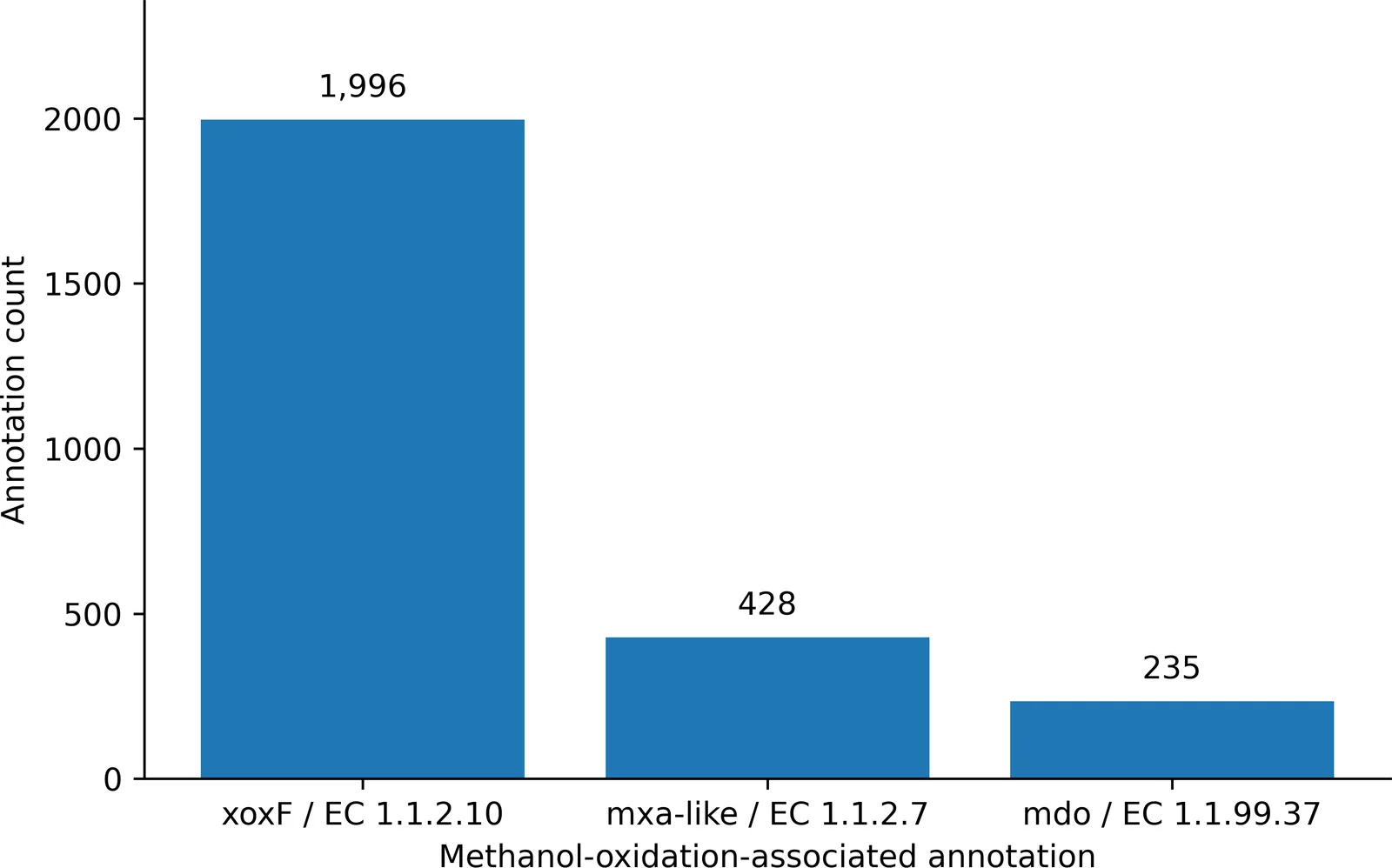
Methanol-oxidation-associated annotation counts in the representative wetland surface sediment co-assembly. Counts are shown for xoxF/EC 1.1.2.10, mxa-like EC 1.1.2.7 methanol dehydrogenase-associated annotations, and mdo/EC 1.1.99.37 annotations recovered from the IMG/M methane-metabolism output for the Wetland Surface Sediment combined assembly (3300075153). Counts represent database-derived annotation counts and should be interpreted as community-level functional potential rather than direct evidence of gene activity, enzyme abundance, pathway flux, or ecological dominance.

Earlier environmental studies often emphasized mxaF as a marker for methanol oxidation, but xoxF-centered surveys later showed that reliance on mxaF alone can underestimate methylotrophic diversity and methanol-oxidation potential (6, 7). Mechanistic work further demonstrated that XoxF can function as the predominant methanol dehydrogenase under lanthanide-replete conditions in some organisms, such as *Methylomicrobium buryatense* (4). In that context, the strong representation of xoxF-linked annotations in the wetland surface sediment co-assembly is consistent with the broader view that lanthanide-dependent methanol dehydrogenases can be important components of environmental methanol oxidation potential. However, because xoxF genes can occur in multiple clades and may be present in multiple copies per genome, the higher xoxF annotation count should not be interpreted as a direct measure of ecological dominance, organism abundance, pathway activity, or methanol-oxidation flux.

Taxonomic context from the KEGG-linked organism list further supported the interpretation that the queried annotation space was associated with known C1-metabolizing lineages. The associated organism set included several canonical methanotrophic and methylotrophic genera, including *Methylococcus, Methylomonas, Methylotuvimicrobium, Methylovulum, Methylocaldum, Methylospira, Methyloprofundus,* and *Methylophaga*. At the same time, the list also included broader environmental Proteobacteria such as *Pseudomonas, Marinobacter, Shewanella, Vibrio,* and *Acinetobacter*. This broader organism context is consistent with recent syntheses showing that xoxF-like genes and other rare-earth-element-associated biomolecules occur beyond classical methanotrophic and methylotrophic groups (13). However, the KEGG-linked organism list reflects reference-organism context rather than direct taxonomic assignment of genes from the co-assembly itself. Therefore, we do not assign the 1,996 xoxF-linked annotations, 428 mxa-like annotations, or 235 mdo annotations to specific genera in this data set.

Because wetlands are methane-cycling ecosystems, xoxF-linked methanol-oxidation potential should also be interpreted in relation to upstream methane oxidation. Some xoxF signals in wetland metagenomes may derive from methanotrophs in which methanol oxidation occurs downstream of methane oxidation, while others may derive from methylotrophs or other bacteria. The present annotation-centered analysis does not distinguish these sources, nor does it test linkage to methane monooxygenase markers such as pmoA or mmoX, genomic neighborhood, methanotroph abundance, or redox gradients. Future work integrating xoxF annotations with pmoA/mmoX profiles, contig-level genomic context, MAG-resolved pathway reconstruction, and environmental gradients would help determine how xoxF-linked methanol oxidation is partitioned among methanotrophic and non-methanotrophic community members in wetlands.

Several additional limitations should be considered. First, this study relies on existing IMG/M, EC, and KEGG annotations rather than *de novo* sequence analysis or manual reannotation. Therefore, the results depend on the accuracy and completeness of database annotations and may be affected by misannotation, incomplete annotation, or functional ambiguity. Second, this analysis does not resolve xoxF into clades such as xoxF1, xoxF2, xoxF3, xoxF4, or xoxF5, which can differ in distribution and biochemical properties. Third, metagenomic gene counts represent functional potential rather than transcriptional activity, protein abundance, enzyme activity, or carbon flux. HMM-based validation, curated reference databases, phylogenetic placement, clade-resolved classification, MAG-resolved analysis, transcriptomics, proteomics, and activity-based measurements would be needed to determine the organism-level sources and functional activity of xoxF-linked methanol oxidation in wetland systems. Taken together, our results support wetlands as a relevant setting for xoxF-linked methanol-oxidation potential and motivate more targeted studies of lanthanide-dependent methanol metabolism in wetland methane-cycling communities.

## Supplementary Material

Reviewer comments

## Data Availability

This study used publicly available metagenomic annotation data from the Joint Genome Institute IMG/M database. The representative wetland data set analyzed in this study is the Wetland Surface Sediment combined assembly, IMG/M taxon object ID 3300075153. No new sequence data were generated for this study.

## SUPPLEMENTAL MATERIAL

OPEN PEER REVIEW reviewer-comments.pdf An accounting of the reviewer comments and feedback.

ASM does not own the copyrights to Supplemental Material that may be linked to, or accessed through, an article. The authors have granted ASM a non-exclusive, world-wide license to publish the Supplemental Material files. Please contact the corresponding author directly for reuse.
