# Supplementary material for: xoxF-linked methanol oxidation signals recur across wetland metagenomes
Source: Microbiol Spectr. 2026 Sep 1;14(10):e00889-26. doi: 10.1128/spectrum.00889-26 (PMC13688285; doi:10.1128/spectrum.00889-26)
Supplement: Supplementary file 1 [file reviewer-comments.pdf]

ASM | Microbiology Spectrum

xoxF-linked methanol oxidation signals recur across wetland metagenomes

Sukhjit Kaur and Archana Anand

Corresponding Author(s): Archana Anand, San Francisco State University College of Science and Engineering

Review Timeline:

Submission Date:	March 12, 2026
Editorial Decision:	May 8, 2026
Revision Received:	July 9, 2026
Accepted:	August 11, 2026

Editor: Katharina Kujala

Reviewer(s): Disclosure of reviewer identity is with reference to reviewer comments included in decision letter(s). The following individuals involved in review of your submission have agreed to reveal their identity: Thomas J Smith (Reviewer #1); Muhammad Farhan Ul Haque (Reviewer #2)

Transaction Report:

(Note: With the exception of the correction of typographical or spelling errors that could be a source of ambiguity, letters and reports are not edited. The original formatting of letters and referee reports may not be reflected in this compilation.)

DOI: https://doi.org/10.1128/spectrum.00889-26

Re: Spectrum00889-26 (xoxF-linked methanol oxidation signals recur across wetland metagenomes)

Dear Dr. Archana Anand:

Thank you for the privilege of reviewing your work. Below you will find my comments, instructions from the Spectrum editorial office, and the reviewer comments.

Please return the manuscript within 60 days; if you cannot complete the modification within this time period, please contact me. If you do not wish to modify the manuscript and prefer to submit it to another journal, notify me immediately so that the manuscript may be formally withdrawn from consideration by Spectrum.

Revision Guidelines
To submit your modified manuscript, log into the submission site at https://spectrum.msubmit.net/cgi-bin/main.plex. Go to Author Tasks and click the appropriate manuscript title to begin. The information you entered when you first submitted the paper will be displayed; update this as necessary. Note the following requirements:

• Upload point-by-point responses to the issues raised by the reviewers in a file named "Response to Reviewers," NOT in your cover letter.
• Upload a compare copy of the manuscript (without figures) as a "Marked-Up Manuscript" file.
• Upload a clean .DOC/.DOCX version of the revised manuscript and remove the previous version.
• Each figure must be uploaded as a separate, editable, high-resolution file (TIFF or EPS preferred), and any multipanel figures must be assembled into one file.
• Any supplemental material intended for posting by ASM should be uploaded with their legends separate from the main manuscript. You can combine all supplemental material into one file (preferred) or split it into a maximum of 10 files with all associated legends included.

For complete guidelines on revision requirements, see our Submission and Review Process webpage. Submission of a paper that does not conform to guidelines may delay acceptance of your manuscript.

Data availability: ASM policy requires that data be available to the public upon online posting of the article, so please verify all links to sequence records, if present, and make sure that each number retrieves the full record of the data. If a new accession number is not linked or a link is broken, provide Spectrum production staff with the correct URL for the record. If the accession numbers for new data are not publicly accessible before the expected online posting of the article, publication may be delayed; please contact production staff (Spectrum@asmusa.org) immediately with the expected release date.

Publication Fees: For information on publication fees and which article types are subject to charges, visit ourwebsite. If your manuscript is accepted for publication and any fees apply, you will be contacted separately about payment during the production process; please follow the instructions in that e-mail. Arrangements for payment must be made before your article is published.

ASM Membership: Corresponding authors may join or renew ASM membership to obtain discounts on publication fees. Need to upgrade your membership level? Please contact Customer Service at Service@asmusa.org.

The ASM Journals program strives for constant improvement in our submission and publication process. Please tell us how we can improve your experience by taking this quick Author Survey.

Thank you for submitting your paper to Spectrum.

Sincerely,
Katharina Kujala
Editor
Microbiology Spectrum

Reviewer #1 (Comments for the Author):

This in silico environmental metagenomic data analysis study finds that the most commonly found genes likely to be capable of oxidation of methanol to formaldehyde in aerobic methanotrophic and methylotrophic organisms within a number of wetland environments encode enzymes of the lanthanide-dependent XoxF type. This is consistent with results from other genomic and metagenomic studies and is consistent with the view that the relatively recently discovered XoxF methanol dehydrogenases are the most important enzymes in global aerobic methanol oxidation.

I noticed a small number of issues for the Authors' consideration:

1. Lines 57-58. "beyond simple presence-absence patterns" - a few more words of explanation would be helpful.
2. The sentence "One strong wetland comparator....evidence base is still relevantly small" (lines 63-65) is essentially repeated later (lines 68-70) as "One important wetland comparator....how limited the wetland-specific evidence base remains". One of these could be deleted without loss of meaning.
3. Line 99. It would be helpful, here or in the introduction, to add a few words of introduction to the mdo genes and the nicotinoprotein methanol dehydrogenases that they encode.
4. Lines 111-120. It would be interesting to know how many of the putative xoxF genes (and genes of other methanol dehydrogenase types) were associated with each of the genera named here. It would also be relevant to refer to literature describing xoxF-like genes associated with organisms other than methanotrophs and methylotrophs (for example the review by Yang et al. [2025] DOI 10.1093/ismejo/wrae241).
5. Since the study uses sequence annotation information rather than direct biological sequence analysis, it would be appropriate to mention in the Results and Discussion section that the results depend upon the accuracy and completeness of those annotations.

Reviewer #2 (Comments for the Author):

The authors performed a database-driven metagenomic screening of wetland-associated datasets (primarily from IMG/M) to investigate the prevalence of lanthanide-dependent methanol dehydrogenase (xoxF). Using enzyme-centered queries (EC 1.1.2.10) alongside comparisons with mxaF-type methanol dehydrogenases, they report that xoxF-linked annotations are recurrent and dominant in wetland metagenomes. A representative wetland sediment co-assembly showed substantially higher counts of xoxF compared to mxaF and other methanol-oxidation genes, which the authors interpret as evidence that xoxF-associated methanol oxidation is widespread and potentially important in wetlands, albeit at the level of community functional potential rather than organism-resolved metabolism. However, based on these concerns given below, particularly the lack of novelty, overinterpretation of results, and insufficient analytical depth, the manuscript in its current form does not meet the standards required for publication in this journal.

Major comments

• A major concern is the lack of functional specificity of the xoxF signal. xoxF is broadly distributed among methanotrophs, methylotrophs, and other bacteria, and in wetland systems it is frequently associated with methanotrophs where it functions downstream of methane oxidation. Without distinguishing methanotrophic versus non-methanotrophic sources (e.g., via linkage to pmoA/mmoX, genomic context, or MAG-based analysis), the attribution of xoxF abundance specifically to methanol oxidation independent of methane cycling is not justified..

• The manuscript overinterprets gene abundance as evidence of metabolic dominance. Metagenomic gene counts do not directly reflect pathway activity or flux, and the absence of transcriptomic, proteomic, or activity-based validation limits the ability to conclude that xoxF-mediated methanol oxidation is functionally dominant in these environments.

• The comparison between xoxF and mxaF is potentially misleading because xoxF exists as multiple clades (e.g., xoxF1, xoxF2, xoxF3, xoxF4, xoxF5) and often multiple copies per genome, whereas mxaF is typically single-copy. Thus, the reported enrichment may reflect genomic redundancy rather than ecological dominance.

• The study largely re-analyzes previously available wetland metagenomic datasets, and the widespread occurrence of xoxF in environmental systems including wetlands, is already well established. The manuscript does not introduce new datasets, new analytical frameworks, or novel biological insights, limiting its contribution.

• Different xoxF clades have distinct ecological roles and biochemical properties. The study treats xoxF as a single functional entity, missing an opportunity to provide ecologically meaningful insights (e.g., xoxF1, xoxF2, xoxF3, xoxF4, xoxF5 distribution).

• Given wetlands are methane-driven systems, the study does not adequately integrate methanol oxidation with upstream methane oxidation (e.g., correlation with pmoA, methanotroph abundance, or redox gradients). This weakens the ecological interpretation.

• Reliance on EC number/KEGG annotation alone can introduce misannotation or functional ambiguity. There is no validation using hidden Markov models (HMMs), curated databases, or manual curation, which raises concerns about annotation accuracy and specificity.

Response to Reviewers

Manuscript ID: Spectrum00889-26
Title: xoxF-linked methanol oxidation signals recur across wetland metagenomes
Authors: Sukhjit Kaur and Archana Anand

Dear Dr. Kujala and Reviewers,

We sincerely thank the editor and both reviewers for their careful evaluation of our manuscript and for providing constructive suggestions. We are pleased that Reviewer 1 found the study consistent with prior genomic and metagenomic work highlighting the ecological importance of XoxF-type methanol dehydrogenases. We also appreciate Reviewer 2's critical comments, which helped us substantially improve the precision, framing, and limitations of the manuscript.

In the revised manuscript, we have made several broad changes. First, we have softened language that could imply functional activity, flux, or ecological dominance from metagenomic annotation counts alone. Second, we now more explicitly frame the analysis as an annotation-based assessment of community-level functional potential. Third, we have added text acknowledging that xoxF annotations may derive from methanotrophs, methylotrophs, and other bacteria, and that wetland xoxF signals should be interpreted in relation to upstream methane oxidation. Fourth, we have expanded the limitations section to address annotation accuracy, copy-number differences, lack of xoxF clade resolution, and the need for future HMM-based, MAG-resolved, transcriptomic, proteomic, and/or activity-based validation. Finally, we have added discussion of mdo/nicotinoprotein methanol dehydrogenases and incorporated the Yang et al. review recommended by Reviewer 1. We also revised the Figure 1 legend to clarify that the plotted values represent database-derived annotation counts and should be interpreted as community-level functional potential rather than direct evidence of gene activity, enzyme abundance, pathway flux, or ecological dominance.

Below, we respond to each comment point by point.

Reviewer #1

We thank Reviewer 1 for their assessment of the manuscript and for recognizing that our findings are consistent with other genomic and metagenomic studies supporting the importance of XoxF methanol dehydrogenases in aerobic methanol oxidation. We appreciate the specific suggestions, which have helped us improve clarity and balance.

Comment 1

Lines 57-58. "beyond simple presence-absence patterns" - a few more words of explanation would be helpful.

Response: We agree and have revised this sentence to clarify what we meant by this phrase. Rather than implying only whether xoxF is present or absent, we now explain that XoxF clades can differ in distribution, biochemical properties, and likely ecological roles. This revision better connects the sentence to the comparative XoxF literature and prepares the reader for our later acknowledgement that our own analysis does not resolve xoxF clades.

Revision made: We revised the relevant sentence in the Introduction to state that comparative studies indicate that xoxF-rich datasets may contain functionally and ecologically distinct methylotrophic assemblages, rather than a single uniform xoxF-containing group.

Comment 2

The sentence "One strong wetland comparator… evidence base is still relevantly small" is essentially repeated later as "One important wetland comparator… how limited the wetland-specific evidence base remains". One of these could be deleted without loss of meaning.

Response: We agree with the reviewer's comment. We removed the duplicated sentence and retained a single, clearer statement about the Zoige alpine wetland study as an important wetland comparator.

Revision made: The duplicated sentence in the Introduction has been deleted.

Comment 3

Line 99. It would be helpful, here or in the introduction, to add a few words of introduction to the mdo genes and the nicotinoprotein methanol dehydrogenases that they encode.

Response: We agree with the reviewer and have added a brief explanation of mdo/nicotinoprotein methanol dehydrogenases. This helps readers understand why mdo/EC 1.1.99.37 was included as a comparison group alongside xoxF/EC 1.1.2.10 and mxa-like EC 1.1.2.7 annotations.

Revision made: We added text explaining that the mdo category represents nicotinoprotein methanol dehydrogenases, an additional methanol-oxidation-associated enzyme group distinct from classical MxaFI-type and lanthanide-dependent XoxF-type methanol dehydrogenases.

Comment 4

Lines 111-120. It would be interesting to know how many of the putative xoxF genes, and genes of other methanol dehydrogenase types, were associated with each of the genera named here. It would also be relevant to refer to literature describing xoxF-like genes associated with organisms other than methanotrophs and methylotrophs, for example the review by Yang et al. (2025), DOI 10.1093/ismejo/wrae241.

Response: We appreciate this suggestion. In the revised manuscript, we clarify that the KEGG-linked organism list used in this analysis reflects reference-organism context for the queried annotation space rather than direct taxonomic assignment of each gene in the co-assembly. Therefore, we cannot accurately assign the 1,996 xoxF/EC 1.1.2.10 genes, 428 mxa-like EC 1.1.2.7 genes, or 235 mdo/EC 1.1.99.37 genes to the named genera without additional contig-level taxonomy, MAG-resolved analysis, or other organism-resolved methods. We have revised the text to avoid implying that the gene counts can be attributed directly to those genera.

We also added the Yang et al. review to broaden the discussion beyond canonical methanotrophs and methylotrophs. Specifically, we now note that rare-earth-element-dependent biomolecules, including XoxF-type methanol dehydrogenases and other lanthanide-associated proteins, occur across a broader biological context than classical methane- and methanol-oxidizing taxa alone. This addition supports a more cautious interpretation of the broad organism context recovered in our annotation-centered analysis.

Revision made: We added a sentence citing Yang et al. and revised the taxonomic-context paragraph to emphasize that the organism list is reference-contextual rather than a direct gene-to-genus assignment.

Comment 5

Since the study uses sequence annotation information rather than direct biological sequence analysis, it would be appropriate to mention in the Results and Discussion section that the results depend upon the accuracy and completeness of those annotations.

Response: We agree with the reviewer's comment. We have added this limitation explicitly to the Results and Discussion. We now state that our findings depend on the accuracy and completeness of IMG/M, EC, and KEGG annotations, and that annotation-based screening can introduce misannotation or functional ambiguity. We further note that HMM-based validation, manual curation, clade-resolved XoxF classification, and MAG- or contig-resolved analyses would strengthen future work.

Revision made: A new limitations paragraph has been added to the Results and Discussion section.

Reviewer #2

We thank Reviewer 2 for the detailed and critical assessment of the manuscript. We agree that our original framing could be interpreted as stronger than what an annotation-based metagenomic screen can support. In response, we have substantially revised the manuscript to clarify the scope of inference. The revised manuscript no longer presents xoxF abundance as evidence of metabolic activity, flux, or organism-level dominance. Instead, we frame the finding as the recurrent and comparatively high representation of xoxF-linked annotations in wetland-associated metagenomes, with explicit limitations regarding functional specificity, copy number, xoxF clade resolution, methane-oxidation linkage, and annotation accuracy.

Major Comment 1

A major concern is the lack of functional specificity of the xoxF signal. xoxF is broadly distributed among methanotrophs, methylotrophs, and other bacteria, and in wetland systems it is frequently associated with methanotrophs where it functions downstream of methane oxidation. Without distinguishing methanotrophic versus non-methanotrophic sources, e.g., via linkage to pmoA/mmoX, genomic context, or MAG-based analysis, the attribution of xoxF abundance specifically to methanol oxidation independent of methane cycling is not justified.

Response: We agree with this concern and have revised the manuscript accordingly. Our intent was not to argue that xoxF-linked methanol oxidation is independent of methane cycling. Rather, in wetland systems, xoxF may often occur in methanotrophs where methanol oxidation is downstream of methane oxidation, as well as in methylotrophs and other bacteria. We now state this explicitly.

We have revised the Results and Discussion to avoid attributing xoxF abundance to methanol oxidation independent of methane cycling. We now describe the detected signal as xoxF-linked methanol oxidation potential within a wetland methane-cycling context. We also added a statement that resolving methanotrophic versus non-methanotrophic sources would require pmoA/mmoX linkage, contig-level genomic context, MAG-resolved analysis, or targeted phylogenetic classification, which were beyond the scope of the present annotation-centered screen.

Revision made: We revised the ecological interpretation paragraph to state that xoxF signals in wetlands should be interpreted in relation to methane oxidation and may reflect downstream methanol oxidation in methanotrophs rather than a process independent of methane cycling.

Major Comment 2

The manuscript overinterprets gene abundance as evidence of metabolic dominance. Metagenomic gene counts do not directly reflect pathway activity or flux, and the absence of transcriptomic, proteomic, or activity-based validation limits the ability to conclude that xoxF-mediated methanol oxidation is functionally dominant in these environments.

Response: We agree. We have removed or softened language that could imply metabolic dominance, functional dominance, activity, or flux. We no longer use gene counts to claim that xoxF-mediated methanol oxidation is functionally dominant in wetlands. Instead, we describe xoxF/EC 1.1.2.10 as the

most frequently recovered methanol-oxidation-associated annotation in the queried annotation space of the representative co-assembly.

We have also added explicit language stating that metagenomic gene counts represent functional potential, not transcriptional activity, protein abundance, enzyme activity, or carbon flux. We note that transcriptomic, proteomic, stable-isotope, cultivation-based, or rate-based measurements would be needed to evaluate activity and flux.

Revision made: We changed phrases such as "dominant methanol-oxidation-associated annotation" to "most frequently recovered methanol-oxidation-associated annotation in the queried annotation space" and added a limitations statement distinguishing functional potential from activity or flux.

Major Comment 3

The comparison between xoxF and mxaF is potentially misleading because xoxF exists as multiple clades, e.g., xoxF1, xoxF2, xoxF3, xoxF4, xoxF5, and often multiple copies per genome, whereas mxaF is typically single-copy. Thus, the reported enrichment may reflect genomic redundancy rather than ecological dominance.

Response: We agree and have revised the manuscript to address this limitation. We now explicitly state that xoxF and mxa-like annotations are not directly equivalent as single-copy functional markers. Because xoxF can occur in multiple clades and may be present in multiple copies within genomes, higher xoxF annotation counts may reflect a combination of organism abundance, gene copy-number variation, clade diversity, and genomic redundancy. Therefore, the observed xoxF:mxa-like ratio should not be interpreted as a direct measure of ecological or metabolic dominance.

Revision made: We added a limitation noting that xoxF copy number and clade diversity complicate direct comparison with mxaF/mxaI-like annotations.

Major Comment 4

The study largely re-analyzes previously available wetland metagenomic datasets, and the widespread occurrence of xoxF in environmental systems including wetlands, is already well established. The manuscript does not introduce new datasets, new analytical frameworks, or novel biological insights, limiting its contribution.

Response: We appreciate this critique and have revised the manuscript to better define the contribution. We agree that xoxF occurrence across environmental systems is increasingly well established and that our study does not introduce new sequence datasets. We now frame the contribution more narrowly and explicitly: this manuscript provides a concise wetland-focused synthesis and annotation-centered screening that highlights the repeated recovery and comparatively high representation of xoxF-linked annotations in wetland-associated metagenomic records, a habitat category that remains less foregrounded than marine, freshwater, and other systems in much of the xoxF literature.

We have removed language that could imply discovery of xoxF as a novel environmental phenomenon. Instead, the revised manuscript emphasizes that wetlands are an underexamined and relevant setting for xoxF-linked methanol-oxidation potential, and that our analysis motivates more targeted, organism-resolved and activity-resolved studies.

Revision made: We revised the Introduction and Discussion to clarify that the novelty is not the first detection of xoxF in wetlands, but rather the focused wetland-centered framing and identification of xoxF-linked annotation patterns in wetland-associated IMG/M records.

Major Comment 5

Different xoxF clades have distinct ecological roles and biochemical properties. The study treats xoxF as a single functional entity, missing an opportunity to provide ecologically meaningful insights, e.g., xoxF1, xoxF2, xoxF3, xoxF4, xoxF5 distribution.

Response: We agree that clade-resolved analysis would provide greater ecological resolution. The present analysis was based on EC/KEGG-centered annotation screening and therefore does not resolve xoxF1-xoxF5 clades. We now state this limitation explicitly and avoid treating all xoxF annotations as a single ecologically uniform group.

We also added text noting that future work should use curated XoxF reference alignments, HMM-based searches, and phylogenetic placement to distinguish xoxF clades and evaluate whether wetland xoxF signals are dominated by particular clades.

Revision made: We added language to the Introduction and Discussion acknowledging XoxF clade diversity and identifying clade-resolved classification as an important next step.

Major Comment 6

Given wetlands are methane-driven systems, the study does not adequately integrate methanol oxidation with upstream methane oxidation, e.g., correlation with pmoA, methanotroph abundance, or redox gradients. This weakens the ecological interpretation.

Response: We agree and have revised the manuscript to better integrate methanol oxidation with upstream methane oxidation. We now explicitly state that wetlands are methane-cycling systems and that methanol oxidation may represent a downstream step in methane-derived carbon metabolism, especially in methanotrophs. We also acknowledge that the present analysis does not test correlations between xoxF and pmoA/mmoX, methanotroph abundance, or redox gradients.

To avoid overinterpretation, we now describe the results as a wetland-associated xoxF annotation signal that should be interpreted within the broader methane-cycling context. We identify pmoA/mmoX linkage, redox-stratified sampling, MAG-resolved pathway reconstruction, and transcriptomic/proteomic approaches as priorities for future studies.

Revision made: We added a paragraph in Results and Discussion linking xoxF-associated methanol oxidation potential to upstream methane oxidation and clarifying the limitations of the current dataset.

Major Comment 7

Reliance on EC number/KEGG annotation alone can introduce misannotation or functional ambiguity. There is no validation using hidden Markov models, curated databases, or manual curation, which raises concerns about annotation accuracy and specificity.

Response: We agree. We have added a clear statement that our analysis depends on the accuracy and completeness of IMG/M, EC, and KEGG annotations. We now explicitly acknowledge that annotation-centered screening can introduce false positives, false negatives, or functional ambiguity, particularly for enzyme families with multiple related clades and overlapping annotation labels.

We have also revised the Methods and Discussion to identify HMM-based validation, curated XoxF/MxaF/Mdo reference sets, manual curation, and phylogenetic classification as important future improvements. We have been careful not to present the annotation counts as independently validated gene identities or as direct measures of activity beyond the level supported by the database annotations.

Revision made: We added annotation-dependence language to both Methods and Results/Discussion and softened the interpretation of EC/KEGG-derived counts.

Summary of Major Revisions

1. Removed duplicated language in the Introduction.
2. Clarified the meaning of "beyond simple presence-absence patterns."
3. Added background on mdo/nicotinoprotein methanol dehydrogenases.
4. Added the Yang et al. review on rare-earth-element-dependent biomolecules.
5. Revised taxonomic-context language to clarify that KEGG-linked organism lists do not directly assign genes to genera in the co-assembly.
6. Softened language implying dominance, activity, or flux.
7. Added explicit limitations regarding annotation accuracy, xoxF copy number, clade diversity, lack of HMM validation, lack of MAG-level resolution, and lack of transcriptomic/proteomic/activity validation.
8. Better integrated xoxF-linked methanol oxidation potential with wetland methane cycling and upstream methane oxidation.
9. Revised the Figure 1 legend to clarify that counts represent database-derived annotation counts and community-level functional potential rather than direct evidence of activity, flux, or ecological dominance.

We thank the reviewers again for their constructive comments, which have helped us improve the manuscript's clarity, rigor, and scope of interpretation.

Re: Spectrum00889-26R1 (xoxF-linked methanol oxidation signals recur across wetland metagenomes)

Dear Dr. Archana Anand:

Your manuscript has been accepted, and I am forwarding it to the ASM production staff for publication. Your paper will first be checked to make sure all elements meet the technical requirements. ASM staff will contact you if anything needs to be revised before copyediting and production can begin. Otherwise, you will be notified when your proofs are ready to be viewed.

Data Availability: ASM policy requires that data be available to the public upon online posting of the article, so please verify all links to sequence records, if present, and make sure that each number retrieves the full record of the data. If a new accession number is not linked or a link is broken, provide production staff with the correct URL for the record. If the accession numbers for new data are not publicly accessible before the expected online posting of the article, publication may be delayed; please contact ASM production staff immediately with the expected release date.

Publication Fees: For information on publication fees and which article types have charges, please visit our website. We have partnered with Copyright Clearance Center (CCC) to collect author charges. If fees apply to your paper, you will receive a message from no-reply@copyright.com with further instructions. For questions related to paying charges through RightsLink, please contact CCC at ASM_Support@copyright.com or toll free at +1-877-622-5543. CCC makes every attempt to respond to all emails within 24 hours.

ASM Membership: Corresponding authors may join or renew ASM membership to obtain discounts on publication fees. Need to upgrade your membership level? Please contact Customer Service at Service@asmusa.org.

Embargo Policy: A press release may be issued as soon as the manuscript is posted on the Spectrum Latest Articles webpage. The corresponding author will receive an email with the subject line "ASM Journals Author Services Notification" when the article is available online.

Cover Image Submissions: If you would like to submit a potential Cover Image, please email a file and a short legend to spectrum@asmusa.org. Please note that we can only consider images that (i) the authors created or own and (ii) have not been previously published. By submitting, you agree that the image can be used under the same terms as the published article. Image File requirements: TIF/EPS, 7.5 inches wide by 8.25 inches tall (at least 2,250 pixels wide by 2,475 pixels tall), minimum 300 dpi resolution (600 dpi preferred), RGB, and no figure elements, e.g., arrows or panel labels. The legend should be a short description of the image, 1-2 sentences recommended. Please use the ASM Journals Cover Image Sizing Tool (https://journals.asm.org/pb-assets/html/asm-cover-sizing-tool.html) to ensure that your proposed cover image meets our size requirements.

The ASM Journals program strives for constant improvement in our submission and publication process. Please tell us how we can improve your experience by taking this quick Author Survey.

Thank you for submitting your paper to Spectrum.

Sincerely,
Katharina Kujala
Editor
Microbiology Spectrum

Reviewer #1 (Comments for the Author):

The Authors have carefully attended to all of the issues I raised on the original version, together with a detailed explanation of the changes made.
